# Valence-Specific Modulation in the Accumulation of Perceptual Evidence Prior to Visual Scene Recognition

**DOI:** 10.1371/journal.pone.0038064

**Published:** 2012-05-31

**Authors:** Antonio Schettino, Tom Loeys, Manuela Bossi, Gilles Pourtois

**Affiliations:** 1 Department of Experimental-Clinical and Health Psychology, Ghent University, Ghent, Belgium; 2 Department of Data Analysis, Ghent University, Ghent, Belgium; 3 Department of Psychology, University of Pavia, Pavia, Italy; University of Manchester, United Kingdom

## Abstract

Visual scene recognition is a dynamic process through which incoming sensory information is iteratively compared with predictions regarding the most likely identity of the input stimulus. In this study, we used a novel progressive unfolding task to characterize the accumulation of perceptual evidence prior to scene recognition, and its potential modulation by the emotional valence of these scenes. Our results show that emotional (pleasant and unpleasant) scenes led to slower accumulation of evidence compared to neutral scenes. In addition, when controlling for the potential contribution of non-emotional factors (i.e., familiarity and complexity of the pictures), our results confirm a reliable shift in the accumulation of evidence for pleasant relative to neutral and unpleasant scenes, suggesting a valence-specific effect. These findings indicate that proactive iterations between sensory processing and top-down predictions during scene recognition are reliably influenced by the rapidly extracted (positive) emotional valence of the visual stimuli. We interpret these findings in accordance with the notion of a genuine positivity offset during emotional scene recognition.

## Introduction

Visual object recognition has classically been conceived as resulting from a set of serial computations performed by dedicated ventral object-selective brain regions located in the infero-temporal cortex, eventually enabling to progressively extract the precise meaning of the retinal input [1], [2]. Whereas bottom-up processes are typically emphasized in these hierarchical models, the visual computations performed by these object-selective areas are nonetheless susceptible to top-down modulatory effects, including selective attention [3]–[6], prior expectations [7], [8], contextual information [9], [10], or decision-making [11], [12]. Therefore, visual object recognition processes are not limited to the analysis of sensory information, but they are further shaped by higher order (i.e., not strictly perceptual) processes.

Interestingly, an alternative view has been put forward to account for these complex interaction effects between bottom-up sensory processing and top-down modulatory influences during recognition. Namely, predictive coding models [13]–[21] advocate that visual object recognition processes taking place within the infero-temporal cortex result from the dynamic interplay between (top-down) predictions and (bottom-up) errors [22]. Predictions reflect prior knowledge related to probable events in the sensory environment, and they are employed to reduce the computational burden of visual perception by guiding attention towards salient aspects of the environment, as well as facilitating the interpretation of ambiguous visual input [7]. Whenever a discrepancy is detected between these top-down predictions and bottom-up sensory processing, an error signal (prediction error) is generated and propagated back to higher-level brain regions, with the aim to update or refine the content of the predictions, and in turn accommodate online sensory processing with the current specificities carried by the visual input [14], [19], [21], [23]. In this framework, the expected and actual sensory input are dynamically compared at each stage of processing by means of recursive loops, until the system is able to generate the most likely interpretation of the target object [7], [24].

Of note, asymmetries in speed of processing and visual pathways between low spatial frequency (LSF) and high spatial frequency (HSF) information could potentially provide a mechanistic account to explain predictive coding effects during early stages of recognition of single objects and complex visual scenes [25]. Several studies have already established the differential contribution of LSF vs. HSF input in face recognition [26]–[28], as well as in the processing of complex visual scenes [29]–[32]. More precisely, LSF information seems more useful in identifying the gist of the scene in conditions of fast stimulus presentation (i.e., 30 ms), whereas for longer durations (150 ms) observers rely more on HSF information [30]. Interestingly, because LSF information travels rapidly from early sensory visual areas to prefrontal and anterior temporal regions (via magnocellular projections), this early coarse analysis of the visual input might actually serve to generate predictions about its content [10], [33]–[36].

Nonetheless, all the models reviewed so far have dealt with the processing of neutral visual stimuli, exclusively. Hence, the question remains whether, when encountering emotion-laden objects or scenes, perceptual processes underlying these proactive guesses are comparable to those involved during the processing of neutral stimuli or not. In fact, given the accumulating empirical evidence showing that emotion does not simply add a specific flavor to perception but can have profound influences in stimulus processing, both at the behavioral and neural levels [37]–[42], one can argue that predictive coding during visual scene recognition may reliably be influenced by emotional factors. However, this question has received little empirical support, and it is therefore still unclear whether mechanisms of predictive coding may change during visual scene recognition or not, depending on the extracted emotional content or value of the incoming stimulus. To address this issue, we recently developed and validated a new experimental paradigm. It enables us to study effects of emotion (i.e., valence and/or arousal) on the speed of proactive guesses during scene recognition, both at the behavioral and electrophysiological (event-related brain potentials, ERPs) levels [43]. For each individual trial, participants were presented with series of filtered images that were gradually unfolding the content of a complex visual scene while they had to perform an (orthogonal) animacy judgment task. Each trial began with the presentation of a blurred image, whose content was progressively revealed by increasing, in up to six sequential, parametric and predictive steps, the amount of diagnostic LSF and HSF information. Therefore, this procedure mimicked a “coarse-to-fine” accumulation of perceptual evidence [30], [33], [44]–[46]. Importantly, the visual scenes used in this study (extracted from a standard database) could be neutral, pleasant or unpleasant, based on independent arousal and valence ratings obtained for these visual stimuli. Behavioral results confirmed that this task was suited to study predictive coding effects during scene recognition because participants did not respond randomly, but they accumulated sufficient perceptual evidence before deciding, with high accuracy, whether the content of the scene was living or not [11], [47]–[49]. Importantly, this effect was not identical for the three emotion categories. Participants probably accumulated perceptual evidence less rapidly (reflected in prolonged recognition) for emotional compared to neutral scenes, this effect being most obvious for pictures having a pleasant content. These results could be interpreted as reflecting a negativity bias during scene recognition [50], [51]. Negativity bias refers to the fact that aversive stimuli usually elicit stronger responses compared to appetitive ones, leading in turn to a faster recognition for unpleasant relative to pleasant pictures. However, the prolonged exploration for pleasant scenes was also consistent with the concurrent activation of positivity offset during scene recognition [52], [53]. In this view, when input to the affect system is minimal, positivity may outweigh negativity. Due to their intrinsic hedonistic value, pleasant scenes could therefore be associated with prolonged exploration. Hence, behavioral results of this study [43] were equivocal with regard to the underlying emotional or motivational drive accounting for these findings.

Whereas these results shed light on mechanisms underlying the generation of proactive guesses during scene recognition and how emotion may influence these complex processes, a main question also arose regarding the specificity of these effects. For instance, considering the fact that we used an orthogonal task (i.e., animacy judgment), it is conceivable that the emotional content of the scene had little or no direct impact on the expression of processes involved in accumulation of perceptual evidence [54]–[57]. Moreover, it was unclear from these results alone whether emotion as such, or other non-controlled factors, may actually have produced the change in the rate of accumulation of evidence between emotional and neutral scenes. Presumably, the selected visual scenes did not differ only regarding the actual emotional content, but also their intrinsic picture complexity and/or familiarity, even though we took special care to minimize obvious perceptual and structural differences across the three emotion categories [43]. However, if the neutral vs. emotional scenes selected in our study were not properly balanced along these two specific non-emotional dimensions (i.e., picture complexity and familiarity), we cannot exclude the possibility that the reported behavioral effects may be imputed to these factors, rather than the differential processing of the emotional content during scene recognition. Presumably, more complex or less familiar visual scenes might be associated with delayed accumulation of evidence in our task. Hence the question remains whether the prolonged accumulation of evidence found in our study for emotional relative to neutral scenes may (at least partly) be explained by changes in picture complexity and/or familiarity across the three emotion categories, rather than the emotional content per se [43]. Therefore, the goal of the present study was to assess whether trial-by-trial variations along these two dimensions may overshadow or confound genuine effects of emotion during the accumulation of perceptual evidence prior to scene recognition or not.

We referred to picture complexity as the extent to which a target object in the foreground can be easily segregated from its background [58]. Figure-ground segregation is a fundamental process in visual scene recognition [59]–[61]. Following initial sensory registration of contours, the visual system automatically groups regions adjacent to each contour with either the main figure in the foreground or the background, thereby prioritizing, in the subsequent analysis, all regions grouped with the figure [62]. However, despite the ubiquitous importance of this gestalt mechanism in vision, previous research has found only weak correlations between picture complexity (e.g., figure-ground segregation) and visual emotion processing [32], [58], [63], [64]. As a matter of fact, motivationally relevant stimuli, particularly emotional scenes, usually influence late perceptual or even post-perceptual stages of processing, presumably after earlier mechanisms contributing to figure-ground segregation come into play [65], [66]. However, all these studies used (relatively) brief and static presentations of fully detailed neutral vs. emotional stimuli, therefore strongly limiting the online generation of predictions about the actual identity of the incoming visual input. Therefore, these earlier studies did not allow to titrate the potential influence of picture complexity on the accumulation of evidence leading to (emotion) scene recognition. We predicted that, in our experiment, picture complexity might actually influence accumulation of evidence, indicated by slower accumulation rates for pictures characterized by a more complex, as opposed to less complex content (i.e., a less vs. a more obvious figure-ground segregation).

Familiarity, on the other hand, was defined as the frequency of encounter associated with a given stimulus (picture content), following standard practice [67]. Familiarity is a relevant construct to take into account in the present case, given its potential overlap with emotion processes. In fact, novelty has been found to elicit threat-like cardiovascular responses in social situations involving the violation of stereotypical expectations [68]. Moreover, a comparable startle reflex was observed for novel and emotional pictures [69]. These negative evaluations of novel/unfamiliar stimuli could be due to the difficulty with which individuals extract diagnostic information necessary for a quick and efficient recognition [70]. Specifically, high fluency (i.e., enhanced processing facilitation) is accompanied by an increase of positive affective reactions, as evidenced by more positive judgments of neutral pictures presented for a prolonged period of time [71]. This effect could potentially explain well-known psychological phenomena such as “mere-exposure”, that is people's general tendency to prefer stimuli they are repeatedly exposed to [72]–[74]. Accordingly, it is important to establish whether familiarity, rather than emotion (i.e., valence and/or arousal), may account for changes in accumulation of evidence prior to recognition. Given the evidence reviewed here above, we predicted more familiar scenes to be recognized earlier than less familiar scenes in our experiment.

To address these questions, we designed a new experiment based on the previously validated progressive unfolding task [43] and collected data in a sample of healthy adult participants. Noteworthy, in addition to the main memory matching task (old-new judgment; see below), we instructed participants to directly attend to the emotional content of the stimuli by occasionally asking them to rate the emotional valence of the scenes. These instructions are at variance with the animacy judgment task used in our previous study [43]. We reasoned that this manipulation should augment the relevance of emotional features during the task [54], and hence the likelihood to observe reliable differences between the three emotion categories (neutral, pleasant and unpleasant) during accumulation of evidence prior to scene recognition. Furthermore, each and every scene used during the main experiment was subsequently rated in terms of familiarity and picture complexity by two independent samples of participants, using standard 9-point Likert scales. Afterwards, we used these independent ratings in a single-trial analysis to assess whether systematic changes in accumulation of evidence prior to recognition (as measured in the main progressive unfolding experiment) might be confounded by variations along picture complexity and/or familiarity. More specifically, we assessed whether the prolonged exploration for emotional compared to neutral scenes (see results) might be due to systematic changes in picture complexity and/or familiarity across these categories.

## Methods

### Ethics statement

The study was approved by the ethics committee of the Faculty of Psychological and Educational Sciences, Ghent University. All participants were required to give written informed consent prior to their participation.

### Participants

Eighteen psychology students (all women, mean age 21 years, range 18–26) participated in the main experiment (progressive unfolding task) in exchange of 30€. In addition, 20 volunteers (15 women, mean age 23 years, range 18–34) participated in the picture complexity rating experiment, whereas another sample of 21 participants (17 women, mean age 23 years, range 19–37) completed the familiarity rating experiment. Each participant of the two rating experiments received 8€. All individuals were native Dutch speaking, right-handed, had normal or corrected-to-normal vision, with no history of neurological or psychiatric disorders.

### Stimuli

The visual stimuli were selected from the International Affective Picture System (IAPS) [75], a standard database containing neutral and emotionally-evocative pictures depicting objects and scenes across various ecological situations. This database provides normative ratings for the basic dimensions of emotion – including arousal and valence – using the Self-Assessment Manikin (SAM) [76]. The stimulus list consisted of 360 pictures, equally divided into three emotion categories according to their standardized valence scores: neutral, unpleasant and pleasant (Table 1). Notably, these pictures were selected on the basis of mean valence and arousal ratings reported by female responders [75], because only women eventually participated in the main experiment (see above). Since the main purpose was to assess valence-specific effects during scene recognition, the selected pleasant and unpleasant scenes were properly balanced with regard to levels of arousal (see Table 1). Similarly to our previous study [43], highly pleasant (i.e., erotic situations) or highly unpleasant (i.e., mutilations) scenes were not included in the stimulus set, given the specific emotion responses often associated with these two categories [65], [77]. Moreover, we included 16 additional neutral pictures that were only used during the practice session (therefore not considered in the subsequent statistical analyses). Finally, 36 supplementary neutral scenes were scrambled (i.e., each picture was divided into grids of 255×255 pixels, which were randomly shuffled 10 times), thereby disrupting the content of the scene. Thus, a total of 412 IAPS pictures (including practice and scrambled trials) were shown to participants of the main experiment, while participants of the two rating experiments were presented with the 360 main pictures (excluding practice and scrambled scenes).

**Table 1:** Mean values and standard deviations (in parenthesis) of normative valence and arousal scores for the selected IAPS pictures.

Emotion category	Valence	Arousal
Neutral	5.14 (1.38)	3.68 (2.05)
Unpleasant	3.17 (1.61)	4.94 (2.15)
Pleasant	6.95 (1.70)	4.97 (2.30)

Note. Scores range from 1 to 9. Independent samples t-test confirmed a highly significant difference in valence between neutral and unpleasant [t(119) = 29.34, p<.001], neutral and pleasant [t(119) = −26.82, p<.001] and unpleasant and pleasant [t(119) = −52.58, p<.001] scenes. Significant differences were also observed in levels of arousal, specifically between neutral and unpleasant [t(119) = −29.34, p<.001] and neutral and pleasant [t(119) = −30.98, p<.001] pictures. However, no significant arousal difference was evidenced between unpleasant and pleasant scenes [t(119) = −0.77, p = .441], confirming a balanced level of activation between these two emotion conditions.

Number codes of pictures selected from the database [75] are provided, for each category separately. Practice: 2107, 2600, 2980, 5533, 5731, 6837, 7017, 7030, 7036, 7055, 7057, 7140, 7224, 7365, 8121, 8312. Neutral: 1350, 1616, 1675, 1903, 1935, 1947, 2025, 2026, 2034, 2191, 2272, 2273, 2279, 2308, 2357, 2377, 2382, 2383, 2390, 2396, 2445, 2446, 2489, 2495, 2514, 2575, 2579, 2593, 2595, 2597, 2606, 2702, 2720, 2749, 2850, 2880, 4090, 4150, 4220, 4250, 4255, 4274, 4275, 4320, 4325, 4605, 4750, 5040, 5395, 5500, 5531, 5532, 5534, 5535, 5900, 6570.2, 7001, 7002, 7003, 7009, 7011, 7014, 7016, 7018, 7019, 7021, 7032, 7033, 7037, 7038, 7042, 7043, 7044, 7045, 7058, 7061, 7062, 7081, 7096, 7130, 7160, 7161, 7170, 7180, 7184, 7186, 7188, 7190, 7207, 7236, 7242, 7247, 7248, 7249, 7255, 7287, 7300, 7354, 7484, 7487, 7493, 7500, 7503, 7506, 7512, 7513, 7546, 7547, 7550, 7590, 7595, 7710, 7820, 7830, 8241, 8311, 8325, 9210, 9260, 9700. Unpleasant: 1230, 1240, 1270, 1275, 1280, 1390, 1505, 1617, 1945, 2115, 2130, 2141, 2205, 2276, 2278, 2400, 2455, 2456, 2525, 2681, 2682, 2694, 2695, 2700, 2715, 2716, 2718, 2745.2, 2750, 2752, 2770, 2795, 2799, 2810, 2900.1, 3061, 3160, 3181, 3190, 3210, 3216, 3280, 3300, 3301, 4621, 4635, 4770, 5970, 5973, 6000, 6240, 6241, 6311, 6314, 6561, 6562, 6610, 6800, 6832, 7013, 7023, 7079, 7092, 7136, 7137, 7520, 7521, 8231, 9002, 9005, 9008, 9031, 9041, 9045, 9046, 9080, 9090, 9102, 9145, 9171, 9180, 9182, 9186, 9265, 9270, 9290, 9291, 9295, 9320, 9330, 9331, 9341, 9342, 9390, 9395, 9402, 9404, 9411, 9415, 9417, 9419, 9421, 9435, 9440, 9445, 9469, 9471, 9561, 9584, 9592, 9596, 9635.2, 9830, 9831, 9832, 9912, 9913, 9922, 9926, 9927. Pleasant: 1340, 1463, 1540, 1590, 1595, 1640, 1659, 1660, 1720, 1721, 1811, 1999, 2055.2, 2056, 2092, 2151, 2156, 2158, 2224, 2274, 2300, 2331, 2344, 2346, 2352, 2398, 2605, 2616, 2655, 3005.2, 4500, 4530, 4534, 4536, 4559, 4571, 4600, 4601, 4603, 4606, 4610, 4612, 4614, 4616, 4617, 4619, 4623, 4624, 4641, 5199, 5215, 5260, 5301, 5480, 5600, 5622, 5628, 5660, 5700, 5814, 5829, 5831, 5849, 5990, 5994, 6250.2, 7200, 7230, 7250, 7260, 7279, 7281, 7282, 7286, 7289, 7291, 7350, 7352, 7390, 7400, 7410, 7430, 7440, 7460, 7461, 7470, 7477, 7481, 7482, 7488, 7489, 7492, 7496, 7501, 7505, 7508, 7515, 7570, 8032, 8050, 8118, 8120, 8162, 8208, 8220, 8280, 8340, 8350, 8371, 8420, 8460, 8461, 8465, 8467, 8497, 8503, 8510, 8531, 8540, 8620; Scrambled: 1112, 1303, 1310, 1645, 1726, 1908, 2002, 2018, 2032, 2038, 2101, 2102, 2104, 2122, 2190, 2220, 2221, 2393, 2440, 2441, 2458, 2480, 2484, 2493, 2506, 2512, 2516, 2518, 2570, 2580, 2635, 2704, 2780, 2830, 2840, 9070.

Each neutral, unpleasant and pleasant scene was arbitrarily paired with another one from the same emotion category based on low-level visual similarities, assessed by systematic visual inspection. More specifically, for each emotion category separately, pictures with a clear distinction between a central figure and a homogeneous background were paired together (e.g., a coffee mug on a table vs. a pocket watch on a dark background), and the same strategy was applied for more complex scenes (e.g., a traffic jam vs. a woman in the crowd). These pairs were used during the main task to minimize the use of purely perceptual, pixel-to-pixel matching strategies (see here below). All the pairs created with this procedure are reported in Table 2.

**Table 2:** Stimulus pairs created for the progressive unfolding task.

	Image pairs					
Pair			Unpleasant		Pleasant	
	First element	Second element	First element	Second element	First element	Second element
1	2191	7513	2455	9180	1640	7286
2	2272	7500	2525	9635.2	1660	4641
3	2308	4250	3300	2752	2158	2156
4	2357	8311	5970	2694	2274	8208
5	2382	7242	5973	9912	2605	7291
6	2390	5535	6000	2115	2616	2300
7	2514	7061	6241	6832	4530	4500
8	2575	2273	6610	6800	4600	2398
9	2579	2595	7013	9926	4616	4610
10	2606	7037	7079	9041	4619	7260
11	2880	7493	7136	9186	4624	7410
12	4090	7003	7137	7092	5260	7440
13	5040	7161	8231	9440	5622	8620
14	5900	6570.2	9080	2715	5831	2056
15	7009	7190	9102	6314	5849	5628
16	7011	4320	9171	2718	5990	7496
17	7014	2377	9182	2456	5994	8120
18	7021	7248	9265	9031	6250.2	8032
19	7038	5532	9290	9320	7200	8510
20	7042	2034	9291	9342	7279	7489
21	7044	7130	9330	9832	7430	7352
22	7045	2396	9395	3181	7460	5480
23	7062	7186	9415	9471	7477	8465
24	7207	7032	9417	6561	7482	8540
25	7287	2026	9421	2900.1	7501	7505
26	7484	7096	9435	7520	7508	5199
27	7503	1350	9584	9469	7570	5814
28	7590	2850	9592	9270	8460	8497
29	7830	7546	9596	2205	8461	2352
30	9260	4275	9831	9402	8503	7470
31	1616	2445	1270	1275	1340	8420
32	1675	2593	1230	2799	1463	8280
33	1903	7255	1240	1617	1540	1595
34	1947	5531	1280	9830	1590	1720
35	2025	7506	1390	2745	1721	8340
36	2446	2383	1505	9002	2224	4606
37	2489	1935	1945	9419	2331	8350
38	2495	2702	2130	9045	2344	1811
39	2720	7033	2141	9090	3005.2	4571
40	2749	4325	2276	2681	4536	2346
41	4150	2597	2682	2795	4559	2055.2
42	4274	7160	2695	9404	4601	7282
43	4750	4255	2716	2700	4603	8162
44	5534	7547	2810	9913	4612	2151
45	7018	9210	3061	6311	4614	7488
46	7019	7300	3160	9005	4617	2092
47	7043	7016	3190	7521	4623	7481
48	7081	7001	3210	6240	5301	8531
49	7170	7002	3216	4770	5600	7350
50	7180	4605	3280	6562	5660	5215
51	7184	7236	4635	9008	7230	1999
52	7188	7820	9445	9927	7250	7461
53	7247	7249	2278	9295	7281	2655
54	7354	7058	2400	9145	7390	5700
55	7487	8325	2770	9341	7492	5829
56	7512	2279	9390	7023	7515	8467
57	7550	4220	9922	9561	8050	4534
58	7595	5395	9046	4621	8118	1659
59	7710	5500	2750	9411	8220	7289
60	8241	9700	3301	9331	8371	7400

Note. These numbers refer to picture codes, as available in the original database [75].

The selected IAPS scenes were resized to 922×691 pixels (90% of the original size) and pre-processed similarly to our previous study [43]: after grayscale conversion, six bandpass spatial frequency filters were applied on every picture (using ImageJ v1.44 software; http://rsb.info.nih.gov/ij/) [78]. As a result, six distinct levels of filtering were obtained for every IAPS scene, each containing a different amount of low and high spatial frequency information [43]. All these modified pictures were finally resized to 768×576 pixels (75% of the original IAPS pictures).

### Procedure

**Figure 1:**
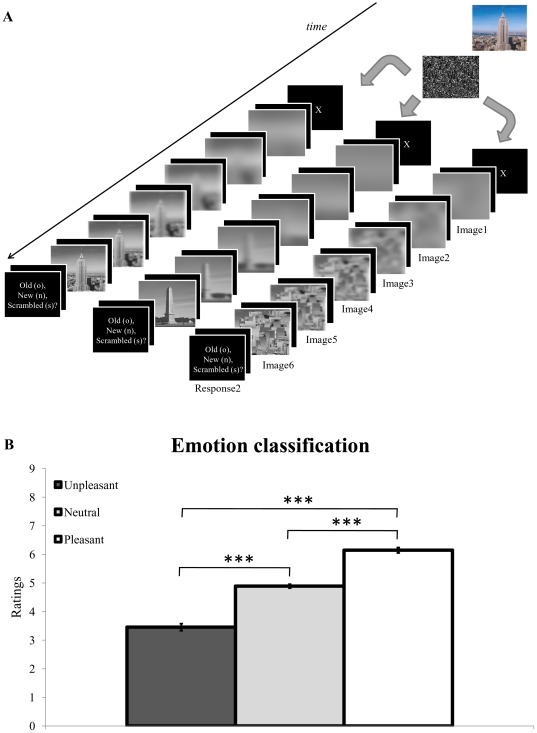
Trial presentation and results of the emotional classification task. (A) Main trial types during the progressive unfolding experiment. A colorful neutral, unpleasant or pleasant IAPS scene (not shown here for copyright reasons) was first presented for 1500 ms, in random order. Following a 2000 ms uniform mask, the same scene (45%), a new one (45%), or a scrambled picture (10%) was progressively revealed in grayscale, using six successive steps varying in a monotonic fashion regarding the content of LSF and HSF information. Each image level was presented for 500 ms, followed by a 250 ms blank screen. Participants had to press a pre-defined button as soon as they could decide whether the gradually unfolded scene was the one seen at the beginning of the trial, a new one, or a scrambled picture (Response1). Five hundred milliseconds after Response1, participants validated their choice and indicated whether the scene was “old”, “new” or “scrambled” by pressing one out of three buttons (Response2). (B) Results of the emotion classification task (occurring after Response2 on 10% of the trials) showed higher scores (corresponding to more pleasant pictures) for pleasant scenes (white bar), followed by neutral (light grey bar) and unpleasant (dark grey bar) scenes. *** p<.001. Vertical bars correspond to standard errors of the means.

Participants were individually tested in a small, dimly lit room, and seated at a viewing distance of 75 cm in front of a 19″ CRT computer screen (refresh rate: 100 Hz). After filling out the informed consent, they were presented with task instructions, followed by a practice block containing 16 neutral pictures. Then, they moved on to the experimental session, divided into twelve blocks, each containing 33 trials. Each trial had the following structure. A colorful, fully detailed picture (922×691 pixels, subtending 18.5°×13.9° of visual angle) was first presented on the screen for 1500 ms, followed by a grayscale mask displayed for 2000 ms. Then, the actual unfolding sequence [43] began. A fixation cross appeared in the center of the screen for 250 ms. The first grayscale, blurred image level of a given picture (768×576 pixels, 15.4°×11.6°) was then presented for 500 ms, followed by a 250 ms blank screen. Next, the second image level of the same picture (identical pixel size, but containing slightly more HSF and LSF information) was displayed for 500 ms, plus the 250 ms blank screen, and the same procedure was repeated until the presentation of the sixth, non-filtered image level. The inter-trial interval was constant and set at 1000 ms (Figure 1A). This experimental manipulation was used to promote a gradual and predictive accumulation of perceptual evidence by progressively adding, in a stepwise fashion, high and low spatial frequency information to the first undistinguishable picture [43]. Importantly, the grayscale and resize conversions relative to the original colorful scene (presented at the beginning of each trial) were applied to discourage participants to use a pixel-to-pixel matching strategy to perform the task. Two separate and consecutive responses were required. First, participants were asked to press a button on a response box (Cedrus RB-730; http://www.cedrus.com/responsepads/rb730.htm) with their right index finger as soon as they felt they gathered enough perceptual evidence to decide, with sufficient confidence, whether the content of the unfolded scene was either the same as the one displayed at the beginning of the trial, a new one, or a new scrambled picture (Response1). These scrambled pictures, for which a separate response was required (see below), were used as “catch” trials to ensure that participants reliably attended to the content of the scenes. Pressing the button immediately interrupted the presentation of the stimulus sequence. After 500 ms, participants were required to perform a three-alternative forced choice delayed matching task, in order to validate their first response (Response1). Specifically, they had to press, on a standard AZERTY keyboard, the “O” key if the unfolded scene was the same as the colorful one previously presented (“old” condition), the “N” key if these two scenes were different (“new” condition), or the “S” key if the unfolded scene was displaying a meaningless content (“scrambled” condition). All these responses, for which no time constraint was established, were coded as Response2. The main purpose of this dual response procedure was to dissociate early visual detection (Response1) from the overt discrimination of the scene requiring a specific stimulus-response mapping (Response2) [43]. Participants were asked to focus on accuracy, but at the same time they were encouraged not to wait until the end of the unfolding sequence to decide about the content of the visual scene (Response1). Responses1 occurring after the presentation of the last/sixth image level were therefore classified as late responses and analyzed separately.

To promote the use of abstract visual representations during overt scene recognition, another experimental manipulation was applied besides the aforementioned inclusion of scrambled pictures as “catch” trials. Specifically, half of the “old” scenes (i.e., unfolded pictures that were identical to the previously encountered colorful scenes) were unpredictably flipped along the horizontal axis between encoding (colorful picture) and retrieval (unfolding). Participants were informed that an “old” response was expected for these “flipped” pictures, since the main task required them to focus on the content of each scene to perform the matching task. In the subsequent behavioral analyses, “old flipped” and “old unflipped” trials were combined into a single “old” condition, to be compared to “new” trials. In sum, for each emotion category (neutral, pleasant, unpleasant), two trial types were contrasted: “old” (N = 180), in which the identity of the initial colorful picture was identical to the scene progressively unfolded, and “new” (N = 180), meaning that the identities of the colorful and unfolded scene were different (although matched as far as possible in terms of low level visual properties using specific stimulus pairs; see the Stimuli section). Hence, for “new” scenes, we used the pairs previously created (see Table 2), with one picture of the pair used as colorful image (encoding) and the other used during unfolding (counterbalanced across participants). We created several stimulus lists such that, across participants, each picture appeared equally often in the “new” and “old” conditions. Importantly, for “new” scenes, no change in terms of emotional content ever occurred between the colorful picture and the scene gradually revealed during unfolding. Accordingly, a neutral colorful picture was always followed by the unfolding of a neutral scene, and the same occurred for emotion-laden stimuli (pleasant-pleasant; unpleasant-unpleasant; see also Table 2). The order of “old”, “new” and “scrambled” trials was randomized.

Finally, in order to verify whether the emotional content of the selected IAPS pictures was actually perceived as such and in line with the normative ratings [75], as well as to keep the emotional content task-relevant throughout the experiment, participants were occasionally asked, after the registration of Response2, to also rate the emotional valence of the colorful scene presented at the beginning of each trial by means of a standard 9-point SAM [76], with anchor 1 corresponding to “very unpleasant” and anchor 9 to “very pleasant”. This additional emotion classification task concerned 10% of the total number of trials. Such manipulation was also employed to increase the likelihood to detect reliable differences between emotional and neutral scenes during accumulation of evidence prior to scene recognition because, with these specific task demands, participants had to attend to the emotional content of the scene [54], [55].

Stimulus presentation and behavioral response recordings were controlled using E-Prime 2.0. (http://www.pstnet.com/products/e-prime/).

### Rating experiments

Participants were tested in pairs in a dimly lit room, seated at a viewing distance of 75 cm in front of individual 19″ CRT screens. In each pair, one member was assigned to rate familiarity while the other was asked to focus on picture complexity of the pre-selected IAPS scenes. After completing the informed consent, they were presented with task instructions, including examples. Then, they moved on to the experimental session, divided into six blocks of 60 trials, separated by short breaks. After an initial fixation cross displayed for 500 ms, neutral, pleasant and unpleasant colorful pictures (hence corresponding to the picture presented at the beginning of each trial of the main progressive unfolding experiment) were presented on the screen in randomized order for 2000 ms. Participants were asked to ignore the hedonic valence of the scenes and provide either familiarity or picture complexity ratings (depending on the condition they were assigned to) using 9-point Likert scales. In case of familiarity judgments, the question was: “How often have you encountered a scene like the one depicted in the picture?”. Scores ranged from 1 (never) to 9 (very often). Raters judging picture complexity, on the other hand, were presented with the question: “Do you consider this picture as having a homogeneous background and an obvious central figure or do you perceive it as more ‘noisy?’”, with “clear figure-ground” anchoring the lower end of the scale and “complex scene” anchoring the upper end. The visual stimuli were never displayed on the screen during the rating phase.

E-Prime 2.0 was used for stimulus presentation and response recordings.

### Analysis of behavioral data

One-way ANOVAs and post-hoc t-tests were used to verify that the emotional content of the scenes was perceived by our participants in agreement with the normative ratings, as well as to explore differences between neutral, unpleasant and pleasant pictures in terms of familiarity and picture complexity.

Accuracy on the progressive unfolding task was expressed as percentage of correct responses. Moments of recognition (Responses1) across the six image levels were not independent of each other: in fact, perceptual evidence was gradually accumulating based on visual input provided by previous image levels. Therefore, cumulative percentages were calculated. This procedure resulted in six psychometric curves showing the evolution of recognition performance across the six image levels, separately for each memory (old, new) and emotion (neutral, unpleasant, pleasant) condition. To characterize effects of emotion and memory on recognition performance, we used a proportional odds model with memory and emotion as predictors [79]. This complex model provides a regression analysis for ordinal dependent variables (recognition from image level 1,…, recognition from image level 6). This data analysis, performed at the single-trial level, allows to model the cumulative probability up to and including recognition from each image level k (k = 1, …, 5). The derived odds ratio expresses how much the odds of recognition from image level k or earlier is increased (if larger than 1) or decreased (if smaller than 1) across new, old, neutral and emotional (unpleasant and pleasant) contents, and thus provides a single number capturing the shift in psychometric curve. To account for dependencies of trials within the same subject, a multi-level version of the proportional odds model was used, similarly to our previous study [43].

Next, we included the mean scores (averaged across raters) of familiarity and picture complexity obtained for each individual picture as additional predictors in the proportional odds model. We verified whether any effect of emotion and/or memory on recognition performance obtained during the main progressive unfolding experiment could be explained by a concurrent effect of familiarity and/or picture complexity.

The level of significance for all these analyses was set at p<0.05. To control for Type I error, a conservative Bonferroni correction was applied to each of the six pairwise comparisons of interest (i.e., emotion, 3 levels; memory, 2 levels) evaluated in each statistical model for the accuracy.

## Results

### Emotion classification task during the progressive unfolding experiment

Results showed higher ratings for pleasant scenes (M = 6.14, SD = 0.81), followed by neutral (M = 4.89, SD = 0.58) and unpleasant (M = 3.45, SD = 1.06) pictures. A one-way ANOVA on these ratings disclosed a highly significant effect of emotion [F(2, 34) = 39.94, p<.001, η_p_^2^ = .701]. Post-hoc t-tests confirmed highly significant differences between neutral and unpleasant pictures [t(17) = 4.83, p<.001], as well as between neutral and pleasant [t(17) = −7.47, p<.001] and unpleasant and pleasant [t(17) = −6.81, p<.001] scenes (Figure 1B). These results confirmed that participants perceived and identified the emotional content of the pre-selected stimuli in accordance with the published normative ratings [75].

### Accuracy for the progressive unfolding experiment

The percentage of errors remained low in this task (M = 3.66%, SD = 1.85). Likewise, very few errors were committed with “catch” trials (M = 1.75%, SD = 1.90). In addition, the percentage of late responses (Responses1 occurring after the last/sixth image level) was negligible (M = 1.71%, SD = 1.18), providing additional evidence that participants accurately performed the matching task during the gradual stimulus revelation and did not wait until the presentation of the last, fully detailed image level to stop the stimulus sequence (Response1).

**Figure 2:**
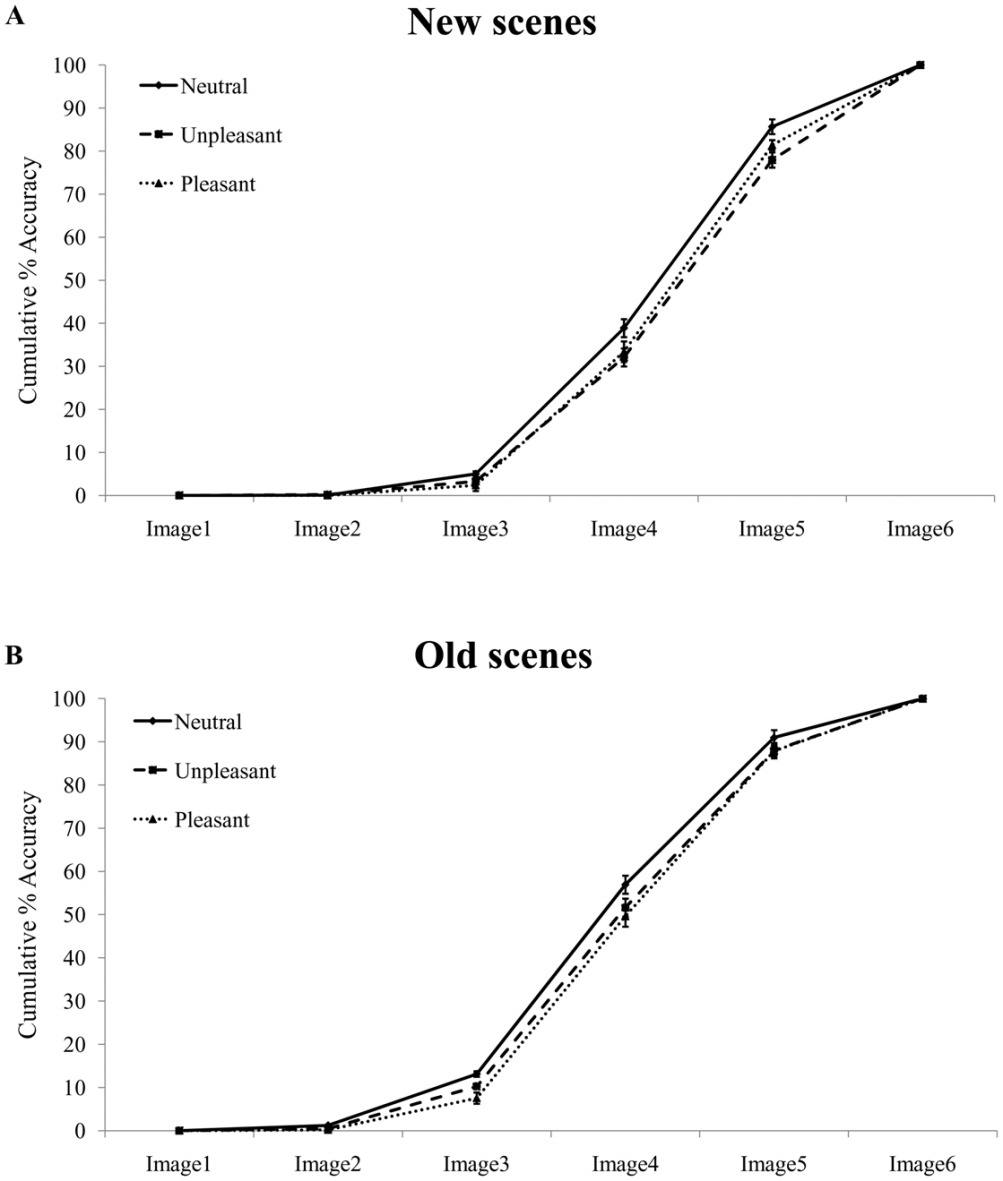
Accuracy in the main progressive unfolding task. Cumulative percentage of correct Responses1 as a function of the six image levels, in the (A) new and (B) old condition, separately for neutral (solid line), unpleasant (dashed line) and pleasant (dotted line) scenes. The shape and variation of the psychometric function according to the main experimental factors (memory and emotion) confirmed that: (i) participants gathered perceptual evidence prior to recognition; (ii) they had a significantly earlier recognition (i.e., less perceptual evidence needed) for old compared to new scenes. Moreover, for each of these two memory levels, emotional scenes led to a delayed recognition relative to neutral scenes. Vertical bars correspond to standard errors of the means.

Table 3 shows the cumulative percentages of correct responses (i.e., Responses1 only when Responses2 were correct). A mixed proportional odds model [43], [79] with memory (old, new) and emotion (neutral, unpleasant, pleasant) as fixed factors, and participant as random effect was carried out on these values, to verify whether the obtained psychometric curves shifted as a function of memory and/or emotion (Figure 2A and 2B). This analysis revealed, as expected, an overall earlier recognition for old compared to new scenes in all emotion conditions (all ps<.001). More interestingly, pairwise comparisons revealed a shift of the distribution as a function of the emotional content of the scenes. Specifically, an earlier recognition (i.e., less accumulation of evidence) was observed when the picture contained a neutral as opposed to an emotional content (all ps<.01), with no significant difference between pleasant and unpleasant scenes (all ps>.05) (see Table 4). The interaction between these two effects (memory and emotion) showed a trend towards significance (p = .064), indicating that the observed delay in recognition for emotional compared to neutral scenes was slightly more pronounced for old relative to new scenes.

**Table 3:** Mean values and standard deviations (in parenthesis) of cumulative percentages of correct responses, separately for each image level, emotion and memory condition.

	New			Old		
Image Level	Neutral	Unpleasant	Pleasant	Neutral	Unpleasant	Pleasant
Image1	0.00 (0.00)	0.00 (0.00)	0.00 (0.00)	0.00 (0.00)	0.00 (0.00)	0.00 (0.00)
Image2	0.10 (0.41)	0.19 (0.79)	0.00 (0.00)	1.22 (2.42)	0.46 (0.00)	0.19 (0.79)
Image3	5.01 (5.36)	3.28 (3.57)	2.35 (3.00)	13.12 (10.40)	10.26 (9.77)	7.55 (8.98)
Image4	38.88 (17.72)	32.07 (17.19)	33.40 (18.24)	56.93 (19.28)	51.62 (16.48)	49.61 (17.68)
Image5	85.66 (14.51)	77.94 (15.87)	81.38 (14.05)	90.98 (8.55)	87.95 (8.70)	87.85 (9.40)
Image6	100.00 (0.00)	100.00 (0.00)	100.00 (0.00)	100.00 (0.00)	100.00 (0.00)	100.00 (0.00)

**Table 4:** Results of the mixed proportional odds model.

Memory condition	Comparison	Odds ratio (95% CI)	p-value
	pleasant vs. neutral	0.75 (0.63,0.90)	0.003*
New	pleasant vs. unpleasant	1.11 (0.92,1.33)	0.246
	unpleasant vs. neutral	0.67 (0.56,0.81)	<0.001*
	pleasant vs. neutral	0.65 (0.53,0.77)	<0.001*
Old	pleasant vs. unpleasant	0.84 (0.71,1.01)	0.064
	unpleasant vs. neutral	0.76 (0.64,0.91)	0.006*

Note. An odds ratio larger than 1 (smaller than 1, respectively) implies that the probability of recognition at earlier levels is higher (smaller, respectively) for the first vs. the second condition included in the comparison. CI indicates confidence interval.

* indicates significant difference after Bonferroni correction.

### Rating experiments

**Figure 3:**
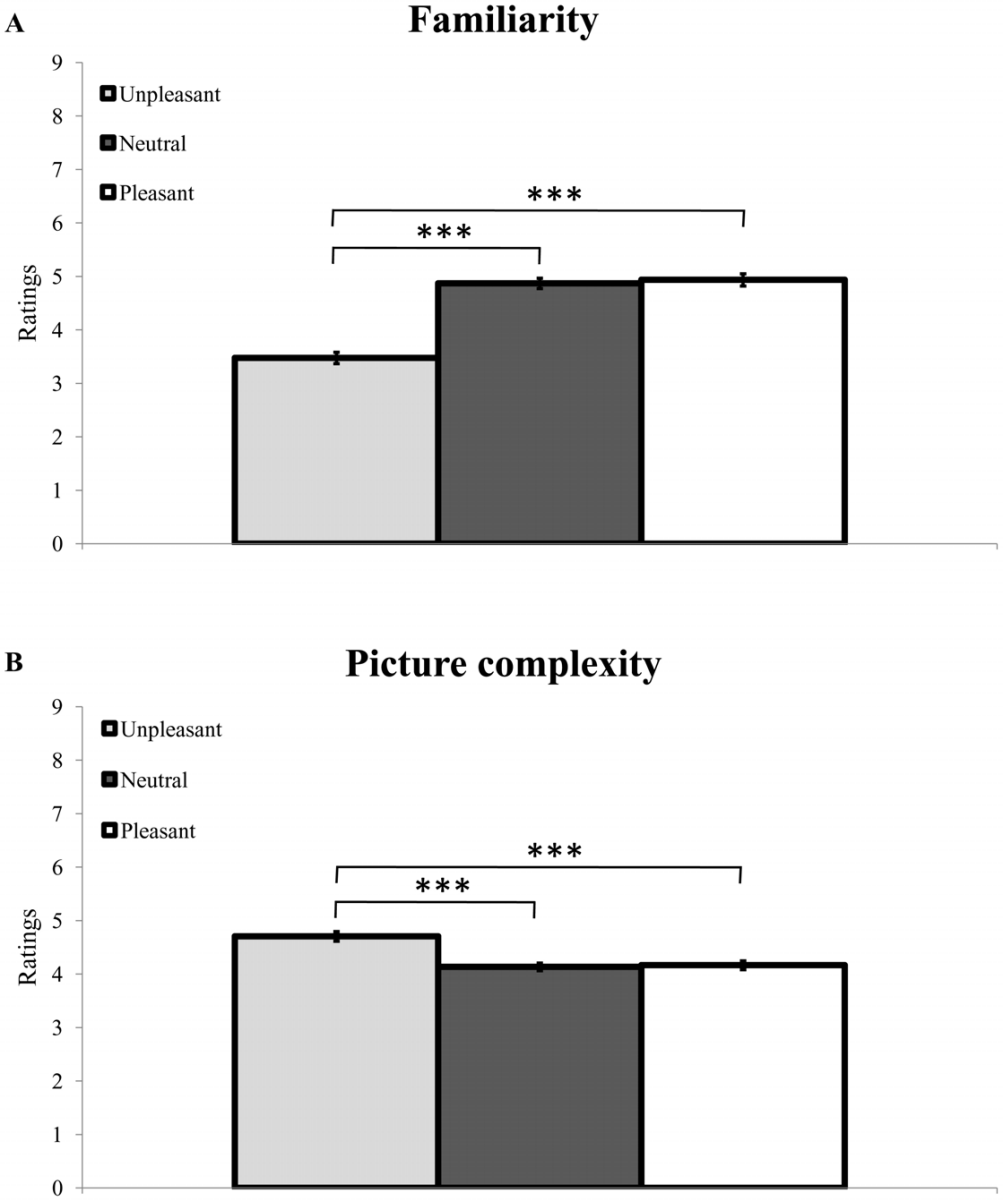
Familiarity and picture complexity ratings. Mean (A) familiarity and (B) picture complexity ratings, separately for neutral (dark grey bar), unpleasant (light grey bar) and pleasant (white bar) scenes. On average, unpleasant scenes were rated as less familiar and perceptually more complex (i.e., less obvious figure-ground segregation) relative to either neutral or pleasant scenes. *** p<.001. Vertical bars correspond to standard errors of the means.

Familiarity ratings of the pre-selected IAPS pictures revealed lower scores for unpleasant scenes (M = 3.48, SD = 0.98), followed by neutral (M = 4.87, SD = 0.89) and pleasant (M = 4.93, SD = 1.05) scenes. A one-way ANOVA on these values disclosed a highly significant effect of emotion [F(2, 40) = 58.64, p<.001, η_p_^2^ = .746]. Post-hoc t-tests showed significant differences between unpleasant and neutral [t(20) = −8.51, p<.001], as well as unpleasant and pleasant [t(20) = −7.70, p<.001] scenes (Figure 3A). Mean familiarity was similar for pleasant and neutral scenes [t(20) = −0.78, p = .445].

Results of picture complexity ratings, on the other hand, showed higher scores for unpleasant (M = 4.70, SD = 0.81), relative to neutral (M = 4.13, SD = 0.64) and pleasant (M = 4.16, SD = 0.74) pictures. A one-way ANOVA carried out on these ratings revealed a highly significant effect of emotion [F(2, 38) = 16.12, p<.001, η_p_^2^ = .459]. Pairwise comparisons confirmed significant differences between unpleasant and neutral [t(19) = 4.19, p<.001] and unpleasant and pleasant [t(19) = 5.87, p<.001] scenes (Figure 3B), whereas no difference was observed between pleasant and neutral scenes [t(19) = −0.28, p = .779]. Thus, unpleasant pictures were characterized by lower familiarity and higher picture complexity compared to neutral and pleasant scenes.

Familiarity and picture complexity were found to be anti-correlated, as confirmed by a significant negative correlation [Pearson's r(360) = −0.40, p<.001].

### Accuracy for progressive unfolding experiment when controlling for familiarity and picture complexity of the visual scenes

Next, we included the average familiarity and picture complexity ratings, obtained for each visual scene separately, as concurrent predictors in the proportional odds model, in order to statistically assess whether the significant effects of memory (i.e., prolonged explorations for new relative to old scenes) and emotion (i.e., prolonged explorations for emotional relative to neutral scenes) might be confounded by trial-to-trial fluctuations along these non-emotional dimensions.

**Figure 4:**
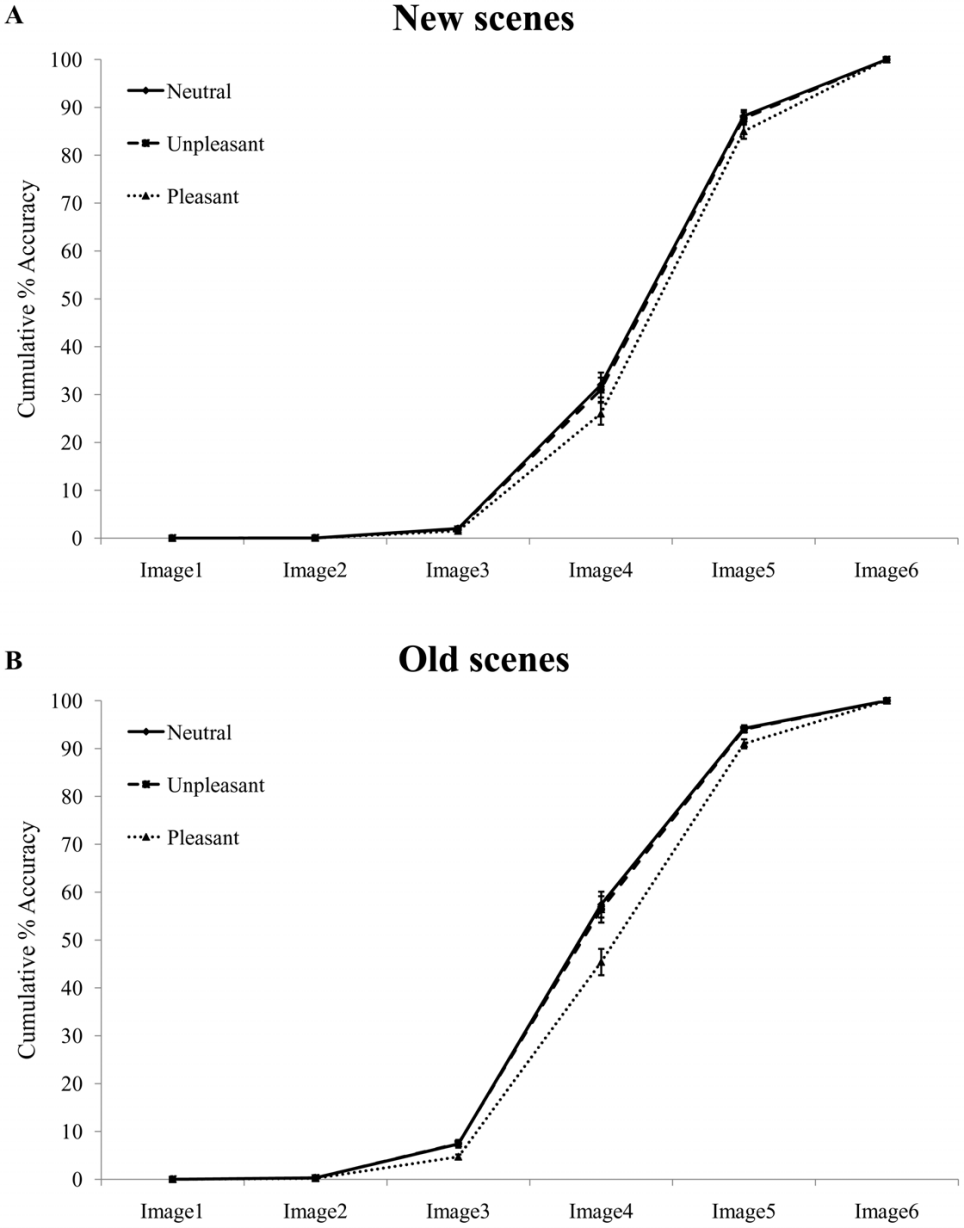
Accuracy in the main progressive unfolding task, adjusted for non-emotional factors. Cumulative percentage of correct Responses1 as a function of the six image levels, in the (A) new and (B) old condition, separately for neutral (solid line), unpleasant (dashed line) and pleasant (dotted line) scenes, once these values were adjusted for familiarity and picture complexity. A significant shift of the psychometric function (corresponding to prolonged accumulation of evidence) was observed for pleasant compared to either neutral or unpleasant scenes, regardless of memory (old vs. new). No significant difference was found between neutral and unpleasant scenes.

Main effects of familiarity and picture complexity were significant (all ps<.001), indicating earlier recognition for more familiar and less complex pictures, in line with our predictions. However, and crucially, the analysis revealed that, after having modeled the potential contribution of these two factors (Table 5), pleasant scenes in the new condition were still associated with a delayed recognition relative to neutral pictures (p = .006) (see also Figure 4A). Pleasant scenes were also recognized later compared to unpleasant pictures (p = .034). However, this difference was no longer considered significant after correction for multiple comparisons (see Table 5). Interestingly, the difference between neutral and unpleasant scenes was no longer significant in this analysis (p = .621), suggesting that familiarity and picture complexity might have accounted for the difference between neutral and emotional scenes in our first analysis (see Table 4). A very similar statistical outcome was observed for old scenes: pleasant pictures led to a prolonged recognition compared to either neutral (p<.001) or unpleasant (p<.001) scenes (see also Figure 4B), whereas the difference between neutral and unpleasant pictures was no longer significant (p = .671). Importantly, the interaction effect between emotion and memory was not significant (p = .102), indicating that the delay in recognition for pleasant scenes was comparable in the new and old conditions. The shift found for pleasant relative to neutral scenes before correcting for complexity and familiarity (see Figure 2) did not therefore appear to be related exclusively to these two specific factors (unlike the case of unpleasant scenes), because the refined analysis controlling for variations along these factors still confirmed this shift (Figure 4).

**Table 5:** Results of the alternative mixed proportional odds model, once item-specific values along familiarity and picture complexity were included in the model.

		Familiarity and picture complexity		Familiarity alone		Picture complexity alone	
Memory condition	Comparison	Odds ratio (95% CI)	p-value	Odds ratio (95% CI)	p-value	Odds ratio (95% CI)	p-value
New	pleasant vs. neutral	0.76 (0.63,0.91)	0.006*	0.74 (0.62,0.88)	0.002*	0.76 (0.63,0.91)	0.006*
	pleasant vs. unpleasant	0.80 (0.65,0.98)	0.034	0.88 (0.72,1.07)	0.179	0.76 (0.63,0.92)	0.008
	unpleasant vs. neutral	0.95 (0.78,1.17)	0.621	0.84 (0.69,1.03)	0.092	0.99 (0.81,1.20)	0.939
Old	pleasant vs. neutral	0.62 (0.51,0.74)	<0.001*	0.63 (0.52,0.76)	<0.001*	0.62 (0.51,0.74)	<0.001*
	pleasant vs. unpleasant	0.64 (0.53,0.78)	<0.001*	0.71 (0.58,0.86)	0.001*	0.61 (0.50,0.73)	<0.001*
	unpleasant vs. neutral	0.96 (0.79,1.16)	0.671	0.89 (0.74,1.08)	0.228	1.01 (0.84,1.22)	0.901

Note.

* : significant difference after Bonferroni correction.

In order to assess whether familiarity and picture complexity had different influences on accumulation of evidence processes in our experiment, we next modeled recognition performance separately for familiarity and picture complexity. Including effects of familiarity in the model (Table 5) revealed, in the new condition, a significantly delayed recognition for pleasant relative to neutral scenes (p = .002). The difference between pleasant and unpleasant scenes (p = .179), and between unpleasant and neutral scenes (p = .092) were not significant. In the old condition, pleasant scenes were also recognized reliably later compared to neutral (p<.001) and unpleasant (p = .001) ones, whereas the difference between unpleasant and neutral scenes was not significant (p = .228).

When modeling the specific contribution of picture complexity (Table 5), the analysis revealed, in the new condition, a delayed recognition for pleasant relative to neutral scenes (p = .006), whereas the unpleasant vs. neutral comparison was not significant (p = .939). The difference between recognition of pleasant vs. unpleasant pictures (p = .008) was marginally significant after Bonferroni correction. The analysis of recognition performance in the old condition revealed that pleasant scenes were recognized significantly later relative to neutral (p<.001) and unpleasant (p<.001) scenes, whereas the difference between unpleasant and neutral scenes was not significant (p = .901).

Although these analyses led to the same conclusions, it is interesting to note that – based on the standard Akaike information criterion (AIC) [80] – the model including both familiarity and picture complexity was providing the best statistical fit. More specifically, the AIC was 13488 for the model including only familiarity, 12619 for the model with only picture complexity, and 12615 for the model with both factors. Since a lower AIC value is considered to fit the data better [80], these results suggest that familiarity explained some of the variability over and beyond picture complexity, the inclusion of this latter factor providing a better fit than the former.

## Discussion

The aim of our study was twofold: (i) to investigate whether the emotional valence of complex visual scenes could have an impact on the accumulation of perceptual evidence prior to their recognition, in line with previous findings showing a delayed recognition (i.e., prolonged accumulation of evidence) for emotional compared to neutral stimuli [43]; (ii) to verify whether these effects may be explained by trial-to-trial fluctuations along other non-emotional variables, with a focus on familiarity and picture complexity.

We used a progressive unfolding task that proved to be useful to explore accumulation of evidence processes prior to scene recognition [43]. After a standard picture encoding phase, participants were presented with series of filtered images that were progressively unfolding the same picture content, a new one or a scrambled one relative to encoding, and the task was to decide whether this scene had previously been presented or not (delayed-match-to-sample task). Of note, the content of either the same scene or a new one was progressively revealed by adding up, in a non-linear fashion, LSF and HSF information, providing a “coarse-to-fine” temporal decomposition of the visual stimulus [45], [46], [81]. We reasoned that the use of impoverished LSF information (and HSF information to a lesser extent), largely predominating at the beginning of the unfolding sequence, could foster the generation of proactive guesses about the actual identity of the scene progressively revealed [10], [33], [35].

Results showed a delayed recognition for new compared to old scenes, as well as for emotional relative to neutral pictures, consistent with our previous results [43]. While the former memory effect confirms that participants used abstract visual representations stored in short-term memory to perform the task [82], [83], the latter effect indicates that these predictive coding mechanisms during scene recognition were not immune to the rapidly perceived emotional content of the input stimulus. Specifically, pleasant and unpleasant scenes were associated with a delayed recognition relative to neutral pictures, suggesting an emotion-specific modulation of predictive coding effects during scene recognition. Moreover, this systematic time lag for recognizing emotional scenes was similar in the new and old conditions, suggesting a general effect taking place irrespective of the memory status of the perceived scenes.

However, we also found that familiarity and picture complexity each had a substantial influence on accumulation of evidence processes prior to scene recognition. First, results of the additional rating experiments showed that the selected unpleasant scenes were rated as less familiar than either neutral or pleasant scenes (Figure 3A), consistent with previous work [69], [71]. This result is in line with the well-known “mere-repeated-exposure” phenomenon [72]–[74], showing that human beings tend to develop a preference towards objects deemed familiar. Therefore, unpleasant objects or events that are typically avoided are considered as less familiar, exactly as found in our rating experiment. Second, our results showed that unpleasant pictures were also considered to be perceptually more complex compared to either neutral or pleasant scenes (Figure 3B). More specifically, unpleasant scenes were systematically associated with a less evident figure-ground segregation in the auxiliary rating experiment, an effect which might lead to a decreased fluency to process these scenes and hence confer them a negative valence [70], [71]. Thus, based on the lower familiarity and higher picture complexity scores obtained for the unpleasant relative to the neutral and pleasant scenes selected in our study, one would predict a change in the speed of accumulation of perceptual evidence for this specific class of emotional stimuli, when compared to the two other conditions. Likewise, given the balanced mean ratings for pleasant and neutral scenes, one could anticipate that accumulation of perceptual evidence would be similar for these two conditions. Instead, our single-trial analysis, in which we included familiarity and complexity ratings – obtained for each and every scene separately – as concurrent regressors (in addition to emotion and memory), revealed that pleasant scenes were associated with a distinctive delayed accumulation of evidence relative to the two other conditions, regardless of the memory status (old vs. new) and hence presumably ease of recognition of these scenes. Thus, at first sight, familiarity and complexity ratings alone could not account for the shift obtained for pleasant relative to neutral scenes during the main task. These results provide evidence for the contribution of positivity offset during emotion scene recognition [53], [84]–[86]. This concept refers to the fact that, when inputs to the affect system are minimal, positivity outweighs negativity. As a consequence, organisms may engage in exploratory behavior under conditions in which no immediate threat is detected, with the aim to gain knowledge about novel stimuli in the environment and their potential value, an effect that is usually exacerbated for pleasant/positive compared to neutral or unpleasant stimuli [53]. Accordingly, the results of our study show that participants were prone to gather additional evidence about pictures carrying intrinsic reinforcing hedonistic values (in this case, pleasant pictures), probably because these pictures better matched their actual motivational dispositions. This latter observation also suggests that the influence of positive emotion on perception in our task was probably operating at an abstract level of stimulus representation, before or after specific short-term memory traces came into play. Of note, a prolonged exploration for pleasant relative to neutral or unpleasant scenes in our experiment may alternatively be explained by the differential motivational relevance of this specific emotion stimulus category [53], [84], [87]–[89]. This general account appears unlikely though, because we did not observe any gain or change during accumulation of evidence for unpleasant compared to neutral scenes, despite the obvious motivational and/or evolutionary relevance of these negative stimuli [50], [51].

The prolonged accumulation of evidence for pleasant relative to neutral and unpleasant scenes may stem from an increase in the number of actual iterations made between updated predictions (initially shaped or constrained by the encoding of the scene in short-term memory) and the progressively accumulated degraded sensory evidence during unfolding, with the aim to minimize prediction errors and favor the most likely interpretation concerning the actual identity of the scene [14], [24], [90], [91]. Alternatively, rather than a quantitative change in the ratio between predictions and errors during accumulation of perceptual evidence, the processing of pleasant scenes may be associated with an overall shift in the decision criterion, relative to neutral or unpleasant scenes. In this view, accumulation of sensory evidence would occur equally fast for neutral and unpleasant scenes, but the delayed decision-making process for pleasant scenes would primarily stem from an enhanced competition between (two or more) choices or alternatives at the decision level per se. The use of computational modeling, and more specifically diffusion models, might turn out to be valuable in this context to tease apart these two accounts [11], [48], [92], [93]. According to these models, decision-making is achieved after having accumulated sufficient sensory evidence, and eventually gathered information in favor of one out of two (or more) alternatives, hence reaching a decision threshold [47]–[49]. The speed of accumulation of perceptual evidence (also termed drift rate) heavily depends on the strength of the sensory signal, as well as the signal-to-noise ratio. Thus, the aforementioned computational models provide useful hints to better explain how specific dispositions to engage in exploratory or approach-related behavior in non-threatening environments (i.e., positivity offset) may ultimately influence proactive processes leading to perceptual decision-making. Further studies are needed to assess whether the processing of pleasant scenes is accompanied by a change in the drift rate compared to neutral or unpleasant scenes, or whether genuine post-perceptual processes may account for this emotion effect. Likewise, additional neuroimaging and/or neurophysiological studies might help clarify whether accumulation of evidence processes are actually generic but vary in speed – depending on the emotional content of the input stimulus – or, instead, several non-overlapping accumulation of evidence brain process may co-exist and can be activated predominantly depending on the valence of this input stimulus.

We have to acknowledge some limitations related to our experimental design and specific data analysis. Familiarity and visual complexity ratings of the pre-selected scenes were collected from two independent samples of participants, while another sample of participants completed the unfolding experiment. It would probably have been more optimal, from a statistical point of view, to use a full within-subject design. However, we did not want to create any bias or expectation regarding the content of the pictures that were progressively revealed during the main experiment. Therefore, we could not ask the same participants to rate the pre-selected visual scenes along the familiarity and picture complexity dimensions before the unfolding experiment. Conversely, ratings obtained for these stimuli would probably be influenced by prior exposure and unbalanced explorations during the unfolding experiment, as revealed for pleasant relative to neutral and unpleasant scenes in our study. Another limitation lies in the possible specificity of these effects for women, because we included mainly female participants and a differential processing of the emotional content of visual stimuli for men and women has previously been reported [94]–[96]. However, the pictures were carefully selected according to the normative ratings published in the manual for this specific gender [75]. Moreover, we purposefully decided not to include highly arousing pictures (e.g., mutilations or erotica) in our stimulus set, because these extreme pictures were found to elicit the largest differences between male and female participants in previous research [65], [77].

To sum up, the results of our study show that accumulation of evidence prior to scene recognition is substantially influenced by the perceived emotional content of the visual stimulus. More specifically, emotional scenes were associated with a prolonged accumulation of evidence relative to neutral scenes. Controlling for non-emotional dimensions (i.e., familiarity and picture complexity) further revealed a delayed recognition for pleasant compared to unpleasant and neutral scenes, suggesting a valence-specific influence on the speed of proactive guesses prior to perceptual decision-making. More generally, these findings are consistent with a positivity offset during complex scene recognition. The propensity to dwell longer on pleasant compared to neutral or unpleasant scenes may be explained by a change in the ratio between predictions and errors during accumulation of evidence, while participants actively make guesses and computed online the most probable interpretation regarding the identity of the incoming and progressively unfolded visual scene. Finally, given the evidence showing a strong positivity offset during emotional scene recognition (that cannot easily be accounted for by systematic trial-to-trial fluctuations along familiarity or picture complexity), we believe that this specific experimental paradigm and stimulus set may eventually turn out to be valuable to shed light on possible qualitative alterations during visual emotion perception typically observed in specific psychopathological conditions. For example, this task appears useful to explore possible changes between the expression of positivity offset vs. negativity bias during scene or object recognition, a modification that might characterize exploration strategies preferentially used by depressed or high anxious individuals [97], [98].
